# Economic burden of antimicrobial resistance and inappropriate empiric treatment in Thailand

**DOI:** 10.1017/ash.2023.169

**Published:** 2023-06-29

**Authors:** Thitima Kongnakorn, Eszter Tichy, Kirati Kengkla, Nuttawan Kanokwanvimol, Pichaya Suthipinijtham, Chanyapat Phuripakathorn, Amer Al Taie

**Affiliations:** 1 Modeling and Simulation, Evidera, Bangkok, Thailand; 2 Modeling and Simulation, Evidera, Budapest, Hungary; 3 Division of Clinical Pharmacy, Department of Pharmaceutical Care, School of Pharmaceutical Sciences, University of Phayao, Phayao, Thailand; 4 Center of Health Outcomes Research and Therapeutic Safety (Cohorts), School of Pharmaceutical Sciences, University of Phayao, Phayao, Thailand; 5 Unit of Excellence on Clinical Outcomes Research and Integration (UNICORN), School of Pharmaceutical Sciences, University of Phayao, Phayao, Thailand; 6 Pfizer (Thailand) Limited, Bangkok, Thailand; 7 Pfizer R&D UK Ltd, Tadworth, United Kingdom

## Abstract

**Objective:**

To quantify the economic burden of bacterial antimicrobial resistance in Thailand and estimate potential savings from improving the rate of appropriate empiric treatment, where effective coverage is provided within the first days of infection.

**Design:**

Cost-of-illness study.

**Methods:**

A cost-calculator, decision-tree model was developed using published data and records from 3 Thai hospitals for patients hospitalized with antimicrobial-resistant infections between 2015 and 2019. Direct and indirect costs of antimicrobial-resistant infections were assessed over a 5-year time horizon, with outcomes derived separately for cases having received appropriate empiric treatment versus inappropriate empiric treatment. In a real-world scenario, outcomes were estimated using actual rates of inappropriate empiric treatment, and in a hypothetical scenario, outcomes were estimated using decreased rates of inappropriate empiric treatment.

**Results:**

Over 5 years, in-hospital antimicrobial-resistant infections produced costs of approximately Thai baht (THB) 66.4 billion (USD 2.1 billion) in the real-world scenario and THB 65.8 billion (USD 2.1 billion) in the hypothetical scenario (0.9% cost savings relative to the real-world scenario). Most costs were attributable to income loss due to in-hospital mortality (real world: THB 53.7 billion [USD 1.7 billion]; 80.9% of costs; hypothetical: THB 53.2 billion [USD 1.7 billion]; 80.8% of costs) and hospitalization (real world: THB 10.3 billion [USD 330.8 million]; 15.5% of costs; hypothetical: THB 10.2 billion [USD 328.9 million]; 15.5% of costs).

**Conclusions:**

In-hospital antimicrobial-resistant infections produced a substantial economic toll in Thailand. This public health burden could be reduced with a strategy aimed at decreasing the rate of patients receiving inappropriate empiric treatment.

Antimicrobial resistance (AMR) contributes to substantial morbidity, mortality, and economic costs^1–6^ and has been declared by the World Health Organization as one of the top global public health threats facing humanity.^6,7^ Although AMR is a global issue, the burdens of AMR are greatest in low-resource settings^4^ and the prevalences of resistant bacteria,^8^ resource availability to treat antibiotic-resistant infections,^9^ and antimicrobial prescribing practices^9^ vary across countries and regions.

In Thailand, AMR is considered an urgent health problem with widespread impact.^10,11^ The Thai National Strategic Plan on Antimicrobial Resistance (NSPAR; 2017–2021) was finalized in 2016 as Thailand’s first coordinated national policy to combat AMR.^11^ NSPAR laid out strategies to prevent and control infections, reduce mean antimicrobial consumption, and improve appropriate AMR drug-prescribing practices, aiming to reduce AMR morbidity by 50% and AMR drug consumption in humans by 20% by 2021.^11,12^

As of early September 2022, baseline AMR data for Thailand are still being developed in parallel with an integrated AMR surveillance system.^13^ Consequently, the full extent of the AMR burden in Thailand is unknown, and progress toward NSPAR goals cannot be reliably assessed. Despite implementation of some NSPAR initiatives, effective integrated AMR management in hospitals remains a challenge due to limited national leadership in implementing AMR initiatives, an insufficient number of hospital health professionals with knowledge of AMR, and delays in implementing AMR initiatives due to the COVID-19 pandemic.^14^

Although little is known about the economic impact of AMR in Thailand, available evidence suggests that burdens of AMR-related illness are substantial.^10,15^ In one study based on 2010 data, resistant pathogens were associated with 32% of in-hospital infections and accounted for 71% of in-hospital infection deaths, with AMR-related premature deaths accumulating costs estimated at Thai baht (THB, 2010) 100–107 billion^10^ (USD 3.2–3.5 billion).^16^ THB 100 billion (USD 3.2 million)^16^ was equivalent to 0.9% of Thailand’s 2010 gross domestic product (GDP).^17^

Receiving inappropriate (ie, noncovering) empiric treatment (IAET)^18^ for antimicrobial-resistant bacterial infections, where effective coverage is not provided within the first days of infection, could contribute significantly to the AMR burden by increasing mortality,^18–21^ lengthening hospital stays,^18,19,21,22^ and increasing direct costs^21^ relative to receiving appropriate (ie, covering) empiric treatment (AET). However, few studies have addressed the additional burden imposed by IAET for antimicrobial-resistant infections in Thailand.^23,24^ Two studies reported that educational interventions in Thai hospitals reduced inappropriate antimicrobial prescribing,^23,24^ but these studies combined all types of inappropriate microbial prescribing (eg, prescribing antibiotics for viral infections) and did not specifically address the impact of IAET for bacterial infections.

Economic analyses conducted at a local level are necessary to provide a more realistic and contextualized cost picture of AMR because they can consider localized epidemiological priorities and health service norms.^25^ Evaluations of the cost burden of AMR and the specific effect of IAET in Thailand could fill the knowledge gap on the current situation and may help to inform future antibiotic stewardship in Thailand. We calculated the economic burden of AMR in Thailand and estimated the potential economic impact of decreasing the rate of IAET in resistant infections.

## Methods

### Model overview

A decision-tree model was developed to evaluate the costs of illness for in-hospital antimicrobial-resistant bacterial infections in Thailand. A real-world scenario estimating the actual burden of antimicrobial-resistant infections was compared with a hypothetical scenario estimating how this burden would be reduced with decreased rates of IAET,^20,21^ where more infections would be covered appropriately within several days of infection onset.

The study adopted a societal perspective, considering both direct costs of healthcare resource use and indirect costs of productivity loss due to patient hospitalization and premature mortality. The societal perspective was chosen to understand the impact of AMR on the welfare of the whole population.^26^ The model estimated the burden of in-hospital antimicrobial-resistant infections for the entire population of Thailand over a 5-year time horizon. Model inputs were derived from published studies, Thai national data, and 2015–2019 hospital data obtained directly from collaborators.

In-hospital infections were defined as antimicrobial-resistant infections that were treated in the hospital; the study did not differentiate between hospital-acquired infections, healthcare-associated infections, and community-acquired infections.^27^ AET was defined based on Infectious Diseases Society of America clinical practice guidelines for both the class and duration of antibiotic therapy.^28^ Patients were considered to have received AET if their antibiotic treatment met these guidelines and covered all index pathogens on the index date or ≤2 days later. Appropriate therapy received ≥3 days after the index date was considered IAET.

### Model structure

**Figure 1.**
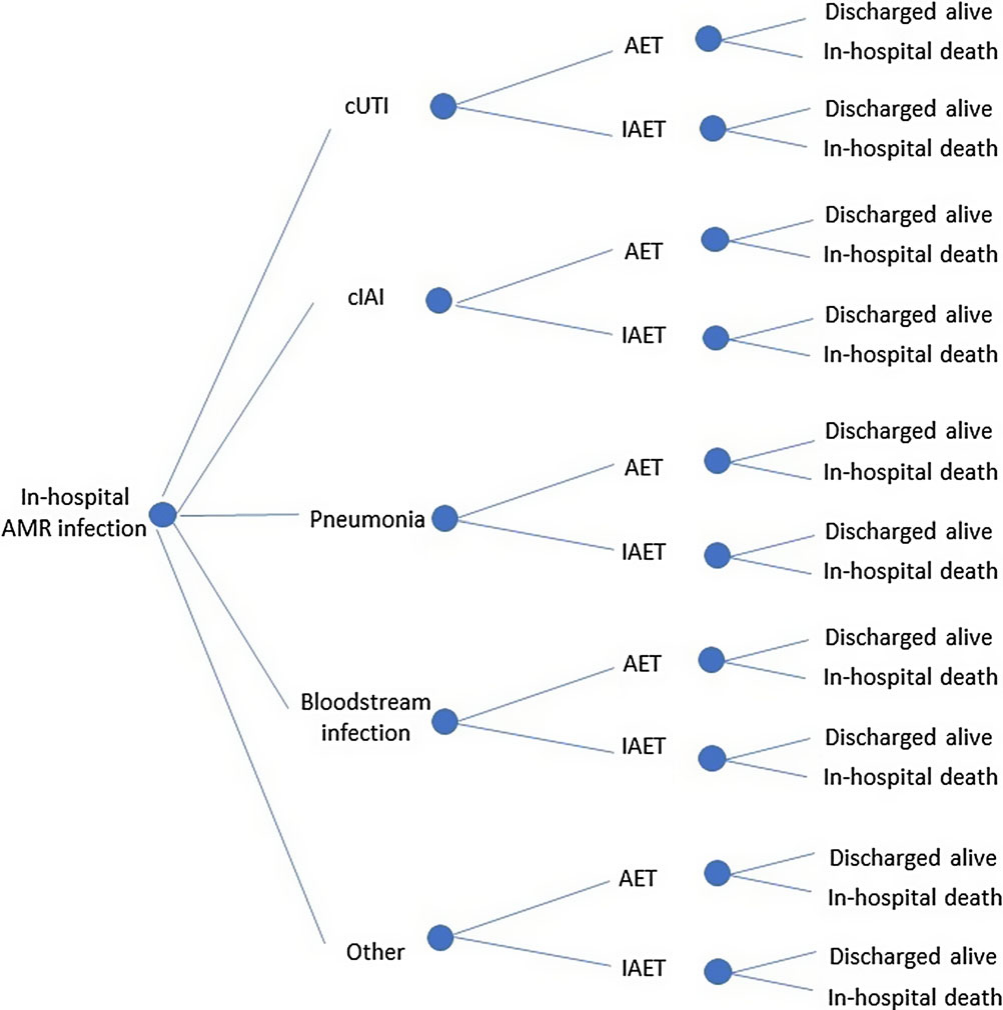
Decision-tree structure. Cases of in-hospital AMR infection were split based on the type of infection, the receipt of AET or IAET, and whether the patient died in hospital or was discharged alive. Note. AET, appropriate empiric treatment; AMR, antimicrobial resistance; cIAI, complicated intra-abdominal infection; cUTI, complicated urinary tract infection; IAET, inappropriate empiric treatment

A decision tree was used to evaluate the economic burden of in-hospital antimicrobial-resistant infections. In the decision tree, cases of in-hospital antimicrobial-resistant infection were first split into 5 branches based on the type of infection, given that cost and clinical outcomes associated with each type of infection differ (Fig. 1). The second decision node divided cases in which empiric treatment provided appropriate coverage within 2–3 days of infection (ie, AET) from those in which empiric treatment did not provide coverage within that timeframe (ie, IAET). The final decision node split cases where the patient was discharged from the hospital alive from those in which the patient died in hospital.

The following economic outcomes were estimated for each case: antibiotics cost, hospitalization cost, income loss due to sick days, and income loss due to premature mortality (for patients who died in hospital). The number of in-hospital deaths was also estimated for each scenario. New patients entered the model every year, and cost outcomes were accumulated for each scenario by infection type and model year. Given the short time horizon, discounting was not included in the model.

### Model population

The model population included patients hospitalized in Thailand with an infection of interest associated with an antimicrobial-resistant pathogen of interest. Infections of interest were complicated urinary tract infections (cUTIs), complicated intra-abdominal infections (cIAIs), pneumonia, bloodstream infections, and surgical-site infections. Resistant pathogens of interest were carbapenem-resistant *Escherichia coli*, carbapenem-resistant *Klebsiella pneumoniae*, carbapenem-resistant *Pseudomonas aeruginosa*, extended-spectrum β-lactamases (ESBL)-producing *E. coli*, and ESBL-producing *K. pneumoniae*. The pathogens of interest were selected based on expert clinical opinions regarding the most prevalent antibiotic-resistant organisms in Thailand.

### Model inputs

#### Epidemiological and clinical inputs

Total hospitalizations in model year 1 reflect the actual number of hospitalizations for all causes in Thailand in 2018 according to Thai National Statistical Office data (Supplementary Table S1).^29^ The number of hospitalizations in subsequent years was increased by 4% per year based on the annual growth in hospitalizations from 2017 to 2018 according to Thai National Statistical Office data.^29^ The number of in-hospital infections for each year was calculated by multiplying the total number of hospitalizations for that year by the proportion of hospitalizations associated with infections (4.4%). This proportion was based on a published estimate from Thai hospital data^30^ and was assumed to remain constant throughout the time horizon.

The number of antimicrobial-resistant infections caused by each pathogen of interest (Table 1) was calculated by multiplying the number of in-hospital infections by the proportion of infections (resistant and nonresistant) caused by each pathogen of interest based on data from Phumart et al (Supplementary Table S1).^10^ The number of infections caused by resistant pathogens was calculated according to data from the 2019 National Antimicrobial Resistance Surveillance Thailand report.^31^ Finally, the number of infections caused by resistant pathogens was stratified by infection type based on the infection distribution recorded in 2015–2019 Thai hospital data.

**Table 1.** Population Values for Number of Infections by Resistant Pathogen

Pathogen	Year 1	Year 2	Year 3	Year 4	Year 5	Source
*E. coli* resistant to carbapenem, n	1,712	1,781	1,852	1,926	2,003	Calculated from^10,29–31^ and hospital data
*K. pneumoniae* resistant to carbapenem, n	10,357	10,772	11,202	11,651	12,117	Calculated from^10,29–31^ and hospital data
*P. aeruginosa* resistant to carbapenem, n	6,237	6,487	6,746	7,016	7,297	Calculated from^10,29–31^ and hospital data
ESBL-producing *E. coli*, n	21,830	22,703	23,612	24,556	25,538	Calculated from^10,29–31^ and hospital data
ESBL-producing *K. pneumoniae*, n	34,769	36,159	37,606	39,110	40,675	Calculated from^10,29–31^ and hospital data

Note. cUTI, complicated urinary tract infection; EBSL, extended-spectrum β-lactamases. Values are summed across infection types (eg, cUTI, pneumonia).

Private data from 3 public Thai hospitals obtained between January 1, 2015, and December 31, 2019 (Supplementary Methods), were used to derive infection distributions, the real-world scenario proportions of patients receiving AET versus IAET, and in-hospital death rates stratified by receipt of AET versus IAET (Table 2).

**Table 2.** Inappropriate Versus Appropriate Empiric Treatment and In-Hospital Death by Infection Type

Variable	cUTI, %	cIAI, %	Pneumonia, %	BSI, %	SSI, %	Source
**Proportion receiving IAET** ^**a**^						
Real world	22.6	33.8	48.8	29.9	22.6	Calculated from hospital data^b^
Hypothetical	21.6	32.8	47.8	28.9	21.6	Calculated^c^
**Proportion dying in hospital** ^**a**^						
AET	9.7	13.0	35.1	22.1	12.5	Calculated from hospital data^b^
IAET	27.8	33.3	49.6	41.4	33.3	Calculated from hospital data^b^

Note. AET, appropriate empiric treatment; cIAI, complicated intra-abdominal infection; cUTI, complicated urinary tract infection; BSI, bloodstream infection; SSI, surgical-site infection; IAET, inappropriate empiric treatment.

a Proportion of patients with bacterial antimicrobial-resistant infections.

b Data on file not publicly available.

c Calculated as real-world proportion receiving IAET minus 1 percentage point.

In the hypothetical scenario, the proportion of patients receiving IAET was decreased by 1 percentage point from the proportion of patients receiving IAET in the real-world scenario. The 1 percentage point decrease was implemented to evaluate the influence of AET versus IAET on AMR costs and allow for simple extrapolation to future cost estimates (ie, to determine cost savings per percentage point).

#### Cost inputs

Cost inputs for antibiotics costs, daily hospitalization costs, and hospital length of stay were based on hospital data collected from 2015–2019 and separated by infection type, receipt of AET or IAET, and whether the patient was discharged alive or died in hospital (Table 3). Cost data were averaged from 2015–2019 costs.

**Table 3.** Direct Cost Inputs

Variable	cUTI	cIAI	Pneumonia	BSI	SSI	Source
**Antibiotic costs per case, THB (USD)^a^**						
**AET**						
Discharged alive	1,405 (45)	3,783 (122)	2,711 (87)	1,715 (55)	3,222 (104)	Hospital data^b^
In-hospital death	2,766 (89)	2,679 (86)	3,088 (99)	2,286 (74)	11,555 (372)	
**IAET**						
Discharged alive	1,945 (63)	6,776 (218)	2,562 (83)	4,365 (141)	2,771 (89)	Hospital data^b^
In-hospital death	6,089 (196)	5,168 (166)	3,753 (121)	3,727 (120)	3,998 (129)	
**Hospitalization costs per case**						
**Daily hospitalization costs, THB (USD)^a^**						
AET	1,033 (33)	1,326 (43)	1,300 (42)	1,129 (36)	1,186 (38)	Hospital data^b^
IAET	1,397 (45)	1,394 (45)	1,342 (43)	1,285 (41)	1,134 (37)	
**Hospital length of stay (days)**						
**AET**						
Discharged alive	12.81	30.75	19.49	10.59	26.00	Hospital data^b^
In-hospital death	20.87	16.68	18.40	13.74	38.54	
**IAET**						
Discharged alive	20.01	28.48	28.79	18.00	32.77	Hospital data^b^
In-hospital death	29.94	46.87	26.91	24.91	43.00	

Note. AET, appropriate empiric treatment; cIAI, complicated intra-abdominal infection; cUTI, complicated urinary tract infection; BSI, bloodstream infection; SSI, surgical-site infection; IAET, inappropriate empiric treatment; THB, Thai baht; USD, United States dollar.

a USD values were converted from THB values using the 2019 annual exchange rate (1 USD = 31.0476 THB).^16^

b Data on file not publicly available.

Direct costs consisted of antibiotics costs and hospitalization costs. Antibiotics costs were input as costs per case, and hospitalization costs were calculated as costs per case by multiplying the relevant daily hospitalization cost by the relevant length of stay.

Indirect costs included in the model were productivity loss due to sick leave and productivity loss due to premature mortality; patients who died in hospital accumulated costs for both types of productivity loss (Table 4). Productivity loss due to sick leave was calculated for each case based on hospital data for hospital length of stay, Thai national employment rates,^32^ and average monthly income based on the 2019 National Statistical Office Labor Survey Report.^32^ Productivity loss due to premature (ie, in-hospital) death was calculated based on hospital data for average patient age and Thai national employment rates,^32^ average monthly income, and average retirement age based on the 2019 National Statistical Office Labor Survey Report.^32^

**Table 4.** Indirect Cost Inputs

Variable	cUTI	cIAI	Pneumonia	BSI	SSI	Source
Proportion of patients employed, %^a^	31	31	31	31	31	^32^
Age of employed patients, average y^a^	46.93	46.93	46.93	46.93	46.93	Hospital data^b^
Retirement age, y^a^	60	60	60	60	60	^32^
Monthly income, THB (USD)^a,c^	26,371 (849)	26,371 (849)	26,371 (849)	26,371 (849)	26,371 (849)	^32^
**Hospitalized days per case**						
*AET*						
Discharged alive	12.81	30.75	19.49	10.59	26.00	Hospital data^b^
In-hospital death	20.87	16.68	18.40	13.74	38.54	
*IAET*						
Discharged alive	20.01	28.48	28.79	18.00	32.77	Hospital data^b^
In-hospital death	29.94	46.87	26.91	24.91	43.00	
**Productivity loss per case, THB (USD)^c^**						
*AET*						
Discharged alive						Calculated
Loss due to sick days	3,144 (101)	7,267 (234)	3,438 (111)	2,242 (72)	6,183 (199)	
In-hospital death						
Loss due to sick days	550 (18)	591 (19)	1,755 (57)	825 (27)	1,309 (42)	
Loss due to premature death	125,850 (4,053)	169,084 (5,446)	455,397 (14,668)	286,731 (9,235)	162,178 (5,224)	
*IAET*						
Discharged alive						Calculated
Loss due to sick days	3,926 (126)	5,163 (166)	3,944 (127)	2,867 (92)	5,938 (191)	
In-hospital death						
Loss due to sick days	2,262 (73)	4,242 (137)	3,628 (117)	2,803 (90)	3,895 (125)	
Loss due to premature death	360,685 (11,617)	432,043 (13,916)	643,524 (20,727)	537,135 (17,300)	432,432 (13,928)	

Note. AET, appropriate empiric treatment; cIAI, complicated intra-abdominal infection; cUTI, complicated urinary tract infection; BSI, bloodstream infection; SSI, surgical-site infection; IAET, inappropriate empiric treatment; THB, Thai baht; USD, United States dollar.

a Assumed the same across all infection types.

b Data on file not publicly available.

c USD values were converted from THB values using the 2019 annual exchange rate (1 USD = 31.0476 THB).^16^

## Results

### Summary

The model estimated that ∼406,000 in-hospital infections caused by resistant pathogens would occur in the real-world scenario over the 5-year time horizon and that these infections would generate total costs of approximately THB 66.4 billion (USD 2.1 billion) (Table 5).^16^ The hypothetical scenario estimated that a 1 percentage point decrease in the proportion of patients with antimicrobial-resistant infections receiving IAET would lead to cost savings of THB 604 million (USD 19.5 million;^16^ 0.9% cost savings) relative to the real-world scenario.

**Table 5.** Estimates of Direct and Indirect Costs^a^

	Real-world scenario costs, THB (USD)^a^	Hypothetical scenario costs, THB (USD)^a^	Costs averted with the hypothetical scenario^b^	
			THB (USD)^a^	%^c^
**Direct costs**				
Antibiotic costs	1,085,560,678 (34,964,399)	1,074,704,088 (34,614,723)	10,856,590 (349,676)	1.0
Hospitalization costs	10,270,404,348 (330,795,435)	10,212,891,329 (328,943,021)	57,513,019 (1,852,414)	0.6
**Indirect costs**				
Productivity loss due to hospitalization	1,335,044,817 (42,999,936)	1,332,465,901 (42,916,873)	2,578,916 (83,063)	0.2
Productivity loss due to premature death^d^	53,722,509,515 (1,730,327,288)	53,189,025,631 (1,713,144,515)	533,483,884 (17,182,774)	1.0
**Total costs**	66,413,519,358 (2,139,087,059)	65,809,086,949 (2,119,619,132)	604,432,409 (19,467,927)	0.9

Note. THB, Thai baht; USD, United States dollar.

a USD values were converted from THB values using the 2019 annual exchange rate (1 USD = 31.0476 THB).^16^

b Calculated by subtracting hypothetical scenario costs from real-world scenario costs.

c Calculated by dividing “costs averted in hypothetical scenario” by real-world scenario costs.

d Includes only in-hospital death.

### Cost outcomes

Most costs in the real-world scenario were associated with income lost due to premature death (80.9% of costs) and direct costs of hospitalization (15.5%) (Table 5). The hypothetical scenario averted costs in all categories, with the greatest cost savings relative to the real-world scenario in averted income loss due to premature mortality (THB 533 million [USD 17.2 million]^16^; 1.0% cost savings) and averted hospitalization costs (THB 57 million [USD 1.9 million]^16^; 0.6% cost savings).

Pneumonia infections accounted for the greatest proportion of costs (71.3% of costs in the real-world scenario), followed by cUTIs (11.4%), bloodstream infections (11.1%), surgical site infections (3.2%), and cIAIs (2.9%) (Supplementary Table S2). The relative decreases in costs for the hypothetical scenario relative to the real-world scenario were similar across infection types, ranging from 0.6% (pneumonia) to 2.0% (cUTI). Costs increased slightly across model years, while the proportion of costs saved in the hypothetical scenario remained the same (Supplementary Table S3).

### Health outcomes

The model estimated that AMR was associated with ∼115,000 in-hospital deaths in the real-world scenario and ∼114,000 in-hospital deaths in the hypothetical scenario, such that the hypothetical scenario averted 0.6% of in-hospital deaths (Supplementary Tables S4 and S5).

## Discussion

This decision-tree model showed that in-hospital antimicrobial-resistant infections impose a substantial economic burden in Thailand, with estimated costs of more than THB 66.4 billion (USD 2.1 billion^16^; 0.41% of 2021 Thailand GDP^33^) over a 5-year period. Decreasing the proportion of patients receiving IAET may help to alleviate this burden; a 1 percentage point decrease in IAET rate (ie, a 1 percentage point improvement) was estimated to reduce the total costs of AMR by 0.9% (THB 604 million [USD 19.5 million]^16^; 0.004% of 2021 Thailand GDP^33^) over 5 years.

Comparison to prior research on the economic burden of AMR in Thailand is limited by a lack of robust data. A 2018 model by Shrestha et al^15^ estimated that direct and indirect economic costs of AMR in Thailand due to resistance to 5 select pathogens totaled USD 0.5 billion (THB 17.6 billion;^34^ 0.12% of 2016 Thailand GDP^35^), although comparison with the present model is limited as the prior model considered both outpatient and inpatient cases and different pathogens of interest. In addition, single-hospital studies based on data from 2008–2012 have estimated an overall societal burden of AMR in Thailand of USD 4.2 billion^36^ and ∼48,000 deaths (2011–2012 data).^37^ A larger-scale study of >1,000 Thai hospitals by Phumart et al^10^ estimated that in-hospital AMR infections in Thailand in 2010 resulted in >38,000 deaths, antibiotic costs of THB 2.5–6.1 billion (USD 81.8–196.0 million),^16^ and indirect costs of THB 40 billion (USD 1.2 billion)^16^ due to premature deaths. After extrapolating from the 1-year time horizon in Phumart et al to the 5-year time horizon in the present study, our model estimated proportionally fewer deaths, lower antibiotic costs, and lower income loss due to premature mortality than in Phumart et al. Differences in mortality and mortality-related income loss may be attributable to improved survival rates and improvement in the treatment landscape over the last decade. Differences in total costs may be due to additional costs included in Phumart et al that are beyond the scope of our model and due to inflation in Thailand.

In the present study, we employed a cost-calculator model that was relatively simple, transparent, and allowed variables to be disaggregated for further analysis. The model benefitted from using clinical and economic inputs specific to Thailand, as well as considering several resistant pathogens of local interest.^31,38,39^ Furthermore, the model provided the first estimation to our knowledge of the potential reduction in economic burden with reduced rates of IAET in Thailand.

This study had several limitations. Some inputs, including hospitalizations and infection rates in model years 2–5, had to be assumed due to lack of sufficient data. Hospital records data were not adjusted for potential biases, including length of stay before infection, comorbidities, or disease severity. In addition, inputs for in-hospital death, rate of IAET, and direct costs were based on data from only 3 Thai hospitals. All included hospitals were public hospitals and were in the northern or central region of Thailand and thus may not be representative of in-hospital death or IAET rates across Thailand. For simplicity, this study did not apply discounting to any costs given the relatively short time horizon. However, productivity loss associated with premature death can be viewed as a future cost; thus, discounting might be considered appropriate. Had we applied 3% discounting to this cost, the cost associated with productivity loss due to premature death would have decreased by THB 11.2 billion (USD 359.4 million)^16^ in the real-world scenario and THB 11.0 billion (USD 355.8 million)^16^ in the hypothetical scenario; savings in the hypothetical scenario would have decreased by THB 111 million (USD 3.6 million).^16^ Interpretation of our analysis should be considered in light of the challenges and potential solutions to decreasing the rates of IAET. Decreasing the rate of IAET may be difficult to implement amid the growing prevalence of AMR and lack of novel antibiotics^40^ for which resistance is not yet prevalent. Implementation of strategies laid out in NSPAR, including improved antimicrobial stewardship, may help reduce the overall prevalence of AMR infections and added costs associated with IAET.

In conclusion, this study provides evidence that in-hospital antimicrobial-resistant infections impose substantial economic impact in Thailand and that decreasing the proportion of cases treated with IAET can help alleviate this burden. Future studies are needed that adjust for potential biases in hospital records data and investigate the impact of IAET in more Thai hospitals.

## Supplementary material

For supplementary material accompanying this paper visit http://doi.org/10.1017/ash.2023.169. click here to view supplementary material

## References

[1] Cosgrove SE. The relationship between antimicrobial resistance and patient outcomes: mortality, length of hospital stay, and health care costs. *Clin Infect Dis* 2006;42 supp 2:S82–S89. 10.1086/499406 16355321

[2] Maragakis LL , Perencevich EN , Cosgrove SE. Clinical and economic burden of antimicrobial resistance. Expert Rev Anti-infect Ther 2008;6:751–763. 18847410 10.1586/14787210.6.5.751

[3] Founou RC , Founou LL , Essack SY. Clinical and economic impact of antibiotic resistance in developing countries: a systematic review and meta-analysis. PLoS One 2017;12:e0189621. 29267306 10.1371/journal.pone.0189621 PMC5739407

[4] Murray CJL , Ikuta KS , Sharara F , et al. Global burden of bacterial antimicrobial resistance in 2019: a systematic analysis. *Lancet* 2022;399:629–655. 10.1016/S0140-6736(21)02724-0 PMC8841637 35065702

[5] Touat M , Opatowski M , Brun-Buisson C , et al. A payer perspective of the hospital inpatient additional care costs of antimicrobial resistance in france: a matched case–control study. Appl Health Econ Health Pol 2019;17:381–389. 10.1007/s40258-018-0451-1 PMC6535148 30506456

[6] Antimicrobial resistance. World Health Organization website. https://www.who.int/news-room/fact-sheets/detail/antimicrobial-resistance. Published 2021. Accessed February 25, 2022.

[7] Ten threats to global health in 2019. World Health Organization website. https://www.who.int/news-room/spotlight/ten-threats-to-global-health-in-2019. Published 2019. Accessed February 25, 2022.

[8] *Antimicrobial Resistance: Global Report on Surveillance.* World Health Organization website. https://apps.who.int/iris/bitstream/handle/10665/112642/?sequence=1. Published 2014. Accessed June 2, 2023.

[9] Pauwels I , Versporten A , Vermeulen H , Vlieghe E , Goossens H. Assessing the impact of the global point prevalence survey of antimicrobial consumption and resistance (Global-PPS) on hospital antimicrobial stewardship programmes: results of a worldwide survey. Antimicrob Resist Infect Control 2021;10:138. 34583775 10.1186/s13756-021-01010-w PMC8478001

[10] Phumart T , Thamlikitkul V , Riewpaiboon A , Prakongsai P , Limwattananon S. Health and economic impacts of antimicrobial resistance in Thailand. J Health Serv Res Pol 2012:352–360.

[11] Thailand’s national strategic plan on antimicrobial resistance 2017–2021. World Health Organization website. https://www.who.int/publications/m/item/thailand-national-strategic-plan-on-antimicrobial-resistance-2017-2021. Accessed February 28, 2022. 10.2471/BLT.20.280644 PMC8381094 34475603

[12] Tangcharoensathien V , Sattayawutthipong W , Kanjanapimai S , Kanpravidth W , Browne, R. , Sommanustweechaia, A. Antimicrobial resistance: from global agenda to national strategic plan, Thailand. Bull World Health Organ 2017;95:599–603. 28804172 10.2471/BLT.16.179648 PMC5537745

[13] Thailand National Government. Midterm progress in the implementation of Thailand’s National Strategic Plan on AMR 2017–2021. Government of Thailand website. https://amrthailand.net/English. Published 2019. Accessed June 2, 2023.

[14] Sumpradit N , Wongkongkathep S , Malathum K , et al. Thailand’s national strategic plan on antimicrobial resistance: progress and challenges. Bull World Health Organ 2021;99:661–673. 34475603 10.2471/BLT.20.280644 PMC8381094

[15] Shrestha P , Cooper BS , Coast J , et al. Enumerating the economic cost of antimicrobial resistance per antibiotic consumed to inform the evaluation of interventions affecting their use. Antimicrob Resist Infect Control 2018;7:98. 30116525 10.1186/s13756-018-0384-3 PMC6085682

[16] 2019 annual exchange rate: Thai baht to US Dollars (1USD = 31.0476 THB). Bank of Thailand website. https://www.bot.or.th/App/BTWS_STAT/statistics/ReportPage.aspx?reportID=123&language=eng. Published 2019. Accessed March 24, 2023.

[17] GDP (current LCU)—Thailand. The World Bank website. https://data.worldbank.org/indicator/NY.GDP.MKTP.CN?end=2010&locations=TH&start=2010. Published 2010. Accessed June 2, 2023.

[18] Davey PG , Marwick C. Appropriate vs. inappropriate antimicrobial therapy. Clin Microbiol Infect 2008;14:15–21. 18318875 10.1111/j.1469-0691.2008.01959.x

[19] Fraser A , Paul M , Almanasreh N , et al. Benefit of appropriate empirical antibiotic treatment: thirty-day mortality and duration of hospital stay. Am J Med 2006;119:970–976. 17071166 10.1016/j.amjmed.2006.03.034

[20] Paul M , Kariv G , Goldberg E , et al. Importance of appropriate empirical antibiotic therapy for methicillin-resistant *Staphylococcus aureus* bacteraemia. J Antimicrob Chemother 2010;65:2658–2665. 20947620 10.1093/jac/dkq373

[21] Zilberberg MD , Nathanson BH , Sulham K , Fan W , Shorr AF. Carbapenem resistance, inappropriate empiric treatment and outcomes among patients hospitalized with Enterobacteriaceae urinary tract infection, pneumonia and sepsis. BMC Infect Dis 2017;17:279. 28415969 10.1186/s12879-017-2383-z PMC5393012

[22] Davey P , Marwick CA , Scott CL , et al. Interventions to improve antibiotic prescribing practices for hospital inpatients. Cochrane Database Syst Rev 2017;2:CD003543. 28178770 10.1002/14651858.CD003543.pub4 PMC6464541

[23] Apisarnthanarak A , Danchaivijitr S , Khawcharoenporn T , et al. Effectiveness of education and an antibiotic-control program in a tertiary-care hospital in Thailand. Clin Infect Dis 2006;42:768–775. 16477551 10.1086/500325

[24] Udomthavornsuk B , Tatsanavivat P , Patjanasoontorn B , et al. Intervention of inappropriate antibiotic use at a university teaching hospital. J Med Assoc Thai 1991;74:429–436. 1797952

[25] Morel CM , Alm RA , Årdal C , et al. A One Health framework to estimate the cost of antimicrobial resistance. Antimicrob Resist Infect Control 2020;9:187. 33243302 10.1186/s13756-020-00822-6 PMC7689633

[26] Byford S , Raftery J. Perspectives in economic evaluation. BMJ 1998;316:1529–1530. 9582152 10.1136/bmj.316.7143.1529 PMC1113167

[27] Cardoso T , Almeida M , Friedman ND , et al. Classification of healthcare-associated infection: a systematic review 10 years after the first proposal. BMC Med 2014;12:40. 24597462 10.1186/1741-7015-12-40 PMC4016612

[28] Tamma PD , Aitken SL , Bonomo RA , Mathers AJ , van Duin D , Clancy CJ. Infectious Diseases Society of America guidance on the treatment of extended-spectrum β-lactamase producing Enterobacterales (ESBL-E), carbapenem-resistant Enterobacterales (CRE), and *Pseudomonas aeruginosa* with difficult-to-treat resistance (DTR-P. *aeruginosa*). Clin Infect Dis 2021;72:1109–1116. 33830222 10.1093/cid/ciab295

[29] Thai National Statistical Office website. http://statbbi.nso.go.th/staticreport/page/sector/th/05.aspx. Accessed March 17, 2022.

[30] Manosuthi W , Thientong V , Moolasart V , Rongrungrueng Y , Sangsajja C , Danchaivijitr S. Healthcare-associated infections at selected hospitals in Thailand. SE Asian J Trop Med Public Health 2017;48:204–212. 29644841

[31] National Antimicrobial Resistance Surveillance, Thailand (NARST) report 2000–2019. Thailand National Government website. http://narst.dmsc.moph.go.th/. Published 2019. Accessed June 2, 2023.

[32] Labour survey report 2019. Thai National Statistical Office website. http://www.nso.go.th/sites/2014en/Pages/survey/Social/Labour.aspx. Updated 2022. Accessed August 1, 2022.

[33] GDP (current LCU)—Thailand. The World Bank website. https://data.worldbank.org/indicator/NY.GDP.MKTP.CN?end=2021&locations=TH&start=2021. Published 2021. Accessed September 7, 2021.

[34] Exchange Rates UK. US dollar to Thai baht spot exchange rates for 2016. Exchange Rates UK website. https://www.exchangerates.org.uk/USD-THB-spot-exchange-rates-history-2016.html. Published 2022. Accessed September 7, 2022.

[35] GDP (current LCU)—Thailand. The World Bank website. https://data.worldbank.org/indicator/NY.GDP.MKTP.CN?end=2016&locations=TH&start=2016. Published 2016. Accessed September 7, 2022.

[36] Phodha T , Riewpaiboon A , Malathum K , Coyte P. Excess annual economic burdens from nosocomial infections caused by multidrug-resistant bacteria in Thailand. Expert Rev Pharmacoecon Outcomes Res 2018;19. 10.1080/14737167.2019.1537123 30321493

[37] Phodha T , Riewpaiboon A , Malathum K , Coyte PC. Annual relative increased in inpatient mortality from antimicrobial resistant nosocomial infections in Thailand. Epidemiol Infect 2019;147:e133. 30868996 10.1017/S0950268818003436 PMC6518492

[38] Chaisathaphol T , Chayakulkeeree M. Epidemiology of infections caused by multidrug-resistant gram-negative bacteria in adult hospitalized patients at Siriraj Hospital. J Med Assoc Thai 2014;97 suppl 3:S35–S45. 24772579

[39] Paveenkittiporn W , Lyman M , Biedron C , et al. Molecular epidemiology of carbapenem-resistant Enterobacterales in Thailand, 2016–2018. Antimicrob Resist Infect Control 2021;10:88. 34090537 10.1186/s13756-021-00950-7 PMC8180034

[40] Lack of new antibiotics threatens global efforts to contain drug-resistant infections. World Health Organization website. https://www.who.int/news/item/17-01-2020-lack-of-new-antibiotics-threatens-global-efforts-to-contain-drug-resistant-infections. Published January 17, 2020. Accessed January 20, 2023.

