# Supplementary material for: Economic burden of antimicrobial resistance and inappropriate empiric treatment in Thailand
Source: Antimicrob Steward Healthc Epidemiol. 2023 Jun 29;3(1):e109. doi: 10.1017/ash.2023.169 (PMC10369446; doi:10.1017/ash.2023.169)
Supplement: Supplementary file 1 [file S2732494X23001699sup001.docx]

# Economic Burden of Antimicrobial Resistance and Inappropriate Empiric Treatment in Thailand

# Appendices

## Supplementary Methods

Private data from three public Thai hospitals (Thammasart Hospital, Maharaj Nakorn Chiangmai Hospital, and Buddhachinaraj Phitsanulok Hospital) obtained between January 1, 2015 and December 31, 2019 were used to derive the real-world scenario proportions of patients receiving AET versus IAET and in-hospital death rates stratified by receipt of AET versus IAET (**Table 2).** Thammasart Hospital is a 439-bed teaching hospital in the Phatum Thani province, located in a region neighboring Bangkok. Maharaj Nakorn Chiangmai Hospital is a 1400-bed teaching hospital in the Chiangmai province, located in a region in Northern Thailand. Buddachinnaraj Phisanulok Hospital is a 922-bed regional hospital in the Phitsanulok province, located in a region in Northern Thailand.

## Supplementary Tables

## TABLE S1. Population values and distributions of infections

|  | Year 1 | Year 2 | Year 3 | Year 4 | Year 5 | Source |
| --- | --- | --- | --- | --- | --- | --- |
| **In-hospital infections** | | | | | | |
| Total hospitalizations in Thailand (n) | 20,519,449 | 21,340,227 | 22,193,836 | 23,081,589 | 24,004,853 | ^1^ |
| % hospitalizations with infection | 4.4% | 4.4% | 4.4% | 4.4% | 4.4% | ^2^ |
| In-hospital infections (n) | 902,856 | 938,970 | 976,529 | 1,015,590 | 1,056,214 | Calculated |
|  | | | | | | |
| **Infections caused by resistant pathogens** | | | | | | |
| ***E. coli* resistant to carbapenem** | | | | | | |
| % infections caused by *E. coli* | 7.1% | 7.1% | 7.1% | 7.1% | 7.1% | ^3^ |
| % *E. coli* infections resistant to carbapenem | 2.7% | 2.7% | 2.7% | 2.7% | 2.7% | ^4^ |
| Infections caused by *E. coli* resistant to carbapenem (n) | 1,712 | 1,781 | 1,852 | 1,926 | 2,003 | Calculated |
| ***K. pneumoniae* resistant to carbapenem** | | | | | | |
| % infections caused by *K. pneumoniae* | 9.7% | 9.7% | 9.7% | 9.7% | 9.7% | ^3^ |
| % *K. pneumoniae* infections resistant to carbapenem | 11.8% | 11.8% | 11.8% | 11.8% | 11.8% | ^4^ |
| Infections caused by *K. pneumoniae* resistant to carbapenem (n) | 10,357 | 10,772 | 11,202 | 11,651 | 12,117 | Calculated |
| ***P. aeruginosa* resistant to carbapenem** | | | | | | |
| % infections caused by *P. aeruginosa* | 3.9% | 3.9% | 3.9% | 3.9% | 3.9% | ^3^ |
| % *P. aeruginosa* resistant to carbapenem | 17.7% | 17.7% | 17.7% | 17.7% | 17.7% | ^4^ |
| Infections caused by *P. aeruginosa* resistant to carbapenem (n) | 6,237 | 6,487 | 6,746 | 7,016 | 7,297 | Calculated |
| **ESBL-producing *E. coli*** | | | | | | |
| % infections caused by ESBL-producing *E. coli* | 7.1% | 7.1% | 7.1% | 7.1% | 7.1% | ^3^ |
| % ESBL-producing *E. coli* | 34.0% | 34.0% | 34.0% | 34.0% | 34.0% | ^4^ |
| Infections caused by ESBL-producing *E. coli* (n) | 21,830 | 22,703 | 23,612 | 24,556 | 25,538 | Calculated |
| **ESBL-producing *K. pneumoniae*** | | | | | | |
| % infections caused by ESBL-producing *K. pneumoniae* | 9.7% | 9.7% | 9.7% | 9.7% | 9.7% | ^3^ |
| % ESBL-producing *K. pneumoniae* | 39.5% | 39.5% | 39.5% | 39.5% | 39.5% | ^4^ |
| Infections caused by ESBL-producing *K. pneumoniae* (n) | 34,769 | 36,159 | 37,606 | 39,110 | 40,675 | Calculated |

Abbreviation: EBSL, extended-spectrum beta-lactamases

## TABLE S2. Costs by infection type

|  | Real-world scenario costs (THB) | Hypothetical scenario costs (THB) | Costs averted with the hypothetical scenario ^a^ | |
| --- | --- | --- | --- | --- |
|  |  |  | **THB** | **%** ^b^ |
| cUTI | 7,591,673,647 | 7,441,106,272 | 150,567,374 | 2.0 |
| cIAI | 1,951,273,383 | 1,928,251,124 | 23,022,259 | 1.2 |
| Pneumonia | 47,385,036,981 | 47,083,536,416 | 301,500,565 | 0.6 |
| Bloodstream infection | 7,349,864,313 | 7,251,416,514 | 98,447,799 | 1.3 |
| Surgical site infection | 2,135,671,034 | 2,104,776,622 | 30,894,412 | 1.4 |
| Total costs | 66,413,519,358 | 65,809,086,949 | 604,432,409 | 0.9 |

Abbreviations: cIAI, complicated intra-abdominal infection; cUTI, complicated urinary tract infection; THB, Thai baht

^a^ Costs averted relative to real-world scenario. Calculated by subtracting hypothetical scenario costs from real-world scenario costs

^b^ Percent of costs averted in hypothetical scenario relative to real-world scenario. Calculated by dividing “costs averted in hypothetical scenario” by real-world scenario costs

## TABLE S3. Costs by model year

|  | Real-world scenario costs (THB) | Hypothetical scenario costs (THB) | Costs averted with the hypothetical scenario ^a^ | | |
| --- | --- | --- | --- | --- | --- |
|  |  |  | **THB** | **%** ^b^ | |
| Year 1 | 12,261,736,376 | 12,150,141,765 | 111,594,611 | | 0.9 |
| Year 2 | 12,752,205,831 | 12,636,147,436 | 116,058,396 | | 0.9 |
| Year 3 | 13,262,294,064 | 13,141,593,333 | 120,700,731 | | 0.9 |
| Year 4 | 13,792,785,827 | 13,667,257,066 | 125,528,761 | | 0.9 |
| Year 5 | 14,344,497,260 | 14,213,947,349 | 130,549,911 | | 0.9 |
| Total costs | 66,413,519,358 | 65,809,086,949 | 604,432,409 | | 0.9 |

Abbreviations: THB, Thai baht

^a^ Costs averted relative to real-world scenario. Calculated by subtracting hypothetical scenario costs from real-world scenario costs

^b^ Percent of costs averted in hypothetical scenario relative to real-world scenario. Calculated by dividing “costs averted in hypothetical scenario” by real-world scenario costs

## TABLE S4. In-hospital deaths by infection type

|  | Real-world scenario deaths (n) | Hypothetical scenario deaths (n) | Deaths averted with the hypothetical scenario ^a^ | | |
| --- | --- | --- | --- | --- | --- |
|  |  |  | **n** | | **%** ^b^ |
| cUTI | 19,008 | 18,759 | 250 | 1.3 | |
| cIAI | 3,365 | 3,331 | 34 | 1.0 | |
| Pneumonia | 72,710 | 72,460 | 250 | 0.3 | |
| Bloodstream infection | 15,451 | 15,344 | 107 | 0.7 | |
| Surgical site infection | 3,979 | 3,931 | 48 | 1.2 | |
| Total deaths | 114,513 | 113,824 | 689 | 0.6 | |

Abbreviations: cIAI, complicated intra-abdominal infection; cUTI, complicated urinary tract infection

^a^ Deaths averted relative to real-world scenario. Calculated by subtracting hypothetical scenario deaths from real-world scenario deaths

^b^ Percent of deaths averted in the hypothetical scenario relative to the real-world scenario. Calculated by dividing “deaths averted in hypothetical scenario” by real-world scenario deaths

## TABLE S5. In-hospital deaths by model year

|  | Real-world scenario deaths (n) | Hypothetical scenario deaths (n) | Deaths averted with the hypothetical scenario ^a^ | |
| --- | --- | --- | --- | --- |
|  |  |  | **n** | **%** ^b^ |
| Year 1 | 21,142 | 21,015 | 127 | 0.6 |
| Year 2 | 21,988 | 21,856 | 132 | 0.6 |
| Year 3 | 22,867 | 22,730 | 138 | 0.6 |
| Year 4 | 23,782 | 23,639 | 143 | 0.6 |
| Year 5 | 24,733 | 24,585 | 149 | 0.6 |
| Total deaths | 114,513 | 113,824 | 689 | 0.6 |

^a^ Deaths averted relative to real-world scenario. Calculated by subtracting hypothetical scenario deaths from real-world scenario deaths

^b^ Percent of deaths averted in hypothetical scenario relative to real-world scenario. Calculated by dividing “deaths averted in hypothetical scenario” by real-world scenario deaths

# References

1. Thai National Statistical Office. <http://statbbi.nso.go.th/staticreport/page/sector/th/05.aspx>. Accessed 2022-17-Mar.

2. Manosuthi W, Thientong V, Moolasart V, Rongrungrueng Y, Sangsajja C, Danchaivijitr S. Healthcare-associated infections at selected hospitals in Thailand. *Southeast Asian J Trop Med Public Health.* 2017;48(1):204-212.

3. Phumart PP, T.; Thamlikitkul, V., Riewpaiboon, A., Prakongsai, P., Limwattananon, S. Health and economic impacts of antimicrobial resistance in Thailand. *Journal of Health Services Research & Policy.* 2012:352-360.

4. Thailand National Government. *National Antimicrobial Resistance Surveillance, Thailand (NARST) report 2000-2019.* 2019.
